# Emulation-Based Dataset EmuIoT-VT for NIDS in IoT Systems

**DOI:** 10.3390/s25165077

**Published:** 2025-08-15

**Authors:** Antanas Čenys, Simran Kaur Hora, Nikolaj Goranin

**Affiliations:** Department of Information Systems, Vilnius Gediminas Technical University, 10223 Vilnius, Lithuania; antanas.cenys@vilniustech.lt (A.Č.); nikolaj.goranin@vilniustech.lt (N.G.)

**Keywords:** Internet of Things, anomaly detection, network intrusion detection, IoT security, IoT dataset, machine learning, deep learning

## Abstract

Due to the rapid expansion of Internet of Things devices and their associated network, security has become a critical concern, necessitating the development of reliable security mechanisms. Anomaly-based NIDS leveraging machine learning and deep learning have emerged as key solutions in detecting abnormal network traffic patterns. However, one challenge that affects the detection rate of machine learning or deep learning-based anomaly NIDS is the class data imbalance present in the existing dataset. Datasets are crucial for the development and evaluation of anomaly-based NIDS for IoT systems. In this study, we introduce EmuIoT-VT, a dataset generated by creating virtual replicas of IoT devices implementing a novel emulation-based method, enabling realistic network traffic generation without relying on any external network emulators. The data was collected in an isolated offline environment to capture clean, uncontaminated network traffic. The EmuIoT-VT is balanced-by-design, containing 28,000 labeled records that are evenly distributed across devices, classes, and subclasses, and supports both binary and multiclass classification tasks. It includes 82 features extracted from raw PCAP data and includes attack categories such as DoS, brute force, reconnaissance, and exploitation. This article presents the novel method and creation of the EmuIoT-VT dataset, detailing data collection, balancing strategy, and details of the dataset structure, and proposes directions for future work.

## 1. Introduction

The Internet of Things (IoT) is a network of physical devices that can be identified uniquely. These devices are embedded with sensors, actuators, and communication interfaces [1]. Nowadays, IoT has become an important part of modern digital ecosystems, as it makes life easier by facilitating connectivity across various domains such as healthcare, transportation, home automation, and industrial operations. IoT devices such as smart locks, voice assistants, connected cameras, and many more devices are becoming popular due to their ability to improve life, save energy, and provide real-time monitoring and intelligent automation without any direct human involvement [2,3,4]. The global IoT market size is estimated at USD 76.97 billion in 2025 and is anticipated to reach around USD 356.23 billion by 2034, accelerating at a compound annual growth rate of 18.56% [5].

However, the rapid adoption of IoT has also introduced some serious security challenges. IoT devices are resource-constrained and lack the processing power to support robust encryption and authentication mechanisms, leaving them susceptible to vulnerabilities [6,7]. Furthermore, due to weak communication protocols, these devices are often exposed to network-layer attacks [8]. The fragmented IoT ecosystem with diverse vendors and protocols results in the implementation of inconsistent security practices [9]. Additionally, embedded third-party software poses major cyberattack risks. For instance, the 2020 Ripple20 vulnerabilities in the Treck TCP/IP stack exposed millions of devices to remote code execution, DoS, and DNS spoofing with minimal attacker effort [10,11].

An IDS is a device or software application that monitors network or system activities. IDS are of two types: network-based and host-based. A host-based IDS monitors operating system events and detects malicious activities that could compromise the host by exploiting its services. However, its scope is restricted to the host that is running IDS. Unlike host-based IDSs, network-based IDSs continuously monitor and observe network traffic for anomalous behavior. Both host- and network-based IDSs use signature-based or anomaly-based detection to detect malicious activity [12].

A signature-based IDS focuses on pattern matching. It detects attacks by matching observed patterns against a database of known attack patterns. It demonstrates high accuracy for known threats; however, it fails to detect zero-day or novel attacks [13]. In contrast, an anomaly-based IDS focuses on outlier detection in legitimate network behavior and flags deviations as potential intrusions. This enables it to detect novel threats without the need for constant updates [14]. In recent years, researchers have increasingly integrated anomaly-based IDSs with artificial intelligence (AI) techniques, especially with machine learning or deep learning techniques, to improve their detection rates [15,16,17,18,19]. However, one challenge that affects the detection rate of machine learning or deep learning-based anomaly NIDS is class imbalance in the dataset. For instance, as highlighted in [20], existing datasets for intrusion detection try to simulate real-network traffic by including more benign traffic samples than the attack samples. This causes the training data to be imbalanced and causes difficulties in detecting certain types of attacks. In [21], the authors found that training NIDS models on imbalanced datasets such as NSL-KDD results in biased learning. Likewise, in [22], the authors emphasized that advanced models such as LSTM fail to learn minority-class attack patterns when trained on imbalanced datasets. In [23], using datasets such as UNSW-NB15 and CICIDS-2017, demonstrated how the anomaly ratio affects algorithm performance in detecting attacks by using two different algorithms: random forests (RF) and support vector machines (SVM). Each algorithm was affected differently by class imbalance. SVMs fail to detect anomalies with acceptable accuracy, whereas RFs seem to be more robust to class imbalance; however, detection deteriorates in extreme anomalies.

The dataset plays an important part in training and evaluating an anomaly-based NIDS that leverages machine learning or deep learning techniques. IoT network intrusion detection datasets for anomaly detection are generally generated using one of three approaches: using a real device, simulation, or emulation. In a real device-based approach, physical IoT hardware is deployed in an actual network environment, capturing traffic from authentic devices. In a simulation-based approach, instead of using real devices or software, virtual nodes of IoT devices and network protocols are modeled using mathematical abstractions or predefined rules. For IoT simulations, tools like Node-RED, OMNeT++, and Cooja are widely used. Emulation serves as a middle ground by using virtual representations of real devices and running software or network stacks in a virtual environment. These emulated devices operate over controlled virtual networks that mimic real-world conditions. For IoT device emulation, tools like CORE, Mininet, QEMU, and GNS-3 are widely used.

While each approach described above has its benefits, they also possess some limitations. Real devices produce authentic traffic but are costly, time-consuming, and difficult to scale consistently. Simulation-based approaches are scalable, but since they rely on abstract models and predefined rules, the network data generated by them is synthetic traffic that may not reflect actual, authentic traffic. Emulation, however, overcomes these issues by allowing real software and protocol stacks to run in a virtualized network. It generates realistic network data without needing physical hardware, making it both cost-effective and accurate.

In this work we propose a novel method that leverages the emulation-based approach to generate realistic IoT network traffic. Leveraging this method, we constructed EmuIoT-VT, a new dataset designed to support machine- or deep-learning-based anomaly detection and address limitations in existing datasets, i.e., class or feature imbalance. The primary objective of proposing this method is to create a virtual replica of an IoT device capable of producing real-network traffic patterns that closely resemble those generated by the actual device under real-world network conditions. Importantly, our focus is not on replicating the complete functional behavior of the physical IoT device (such as a camera capturing videos or a smart clock showing the time and weather forecast or a smart plug switching appliances on/off), but rather on accurately emulating the network behavior of the IoT device. This enables the generation of more realistic network traffic, bridging the gap between controlled experimentation and real-world IoT deployments. The research findings include the following:

- A novel emulation-based method is proposed in this study to generate realistic IoT device network traffic that closely mimics real-world device behavior.
- Through implementation of the proposed novel method, we created a new network traffic dataset, EmuIoT-VT, employing a balanced-by-design strategy.
- The dataset was collected in an isolated offline environment to capture clean raw network traffic.
- To address the issue of class imbalance found in most public IoT datasets, we used a balanced-by-design strategy after network data collection to finalize the dataset.
- The dataset includes 28,000 labeled records evenly distributed across devices, classes, and subclasses, and supports both binary and multiclass classification tasks.
- The dataset provides 82 features extracted from PCAP files and includes attack categories such as DoS, brute force, reconnaissance, and exploitation.

This article is dedicated to the introduction of the novel emulation method and generating the EmuIoT-VT dataset through the implementation of the proposed method. It also provides detailed information about network data collection, dataset balancing strategy, description of the dataset structure, and directions for future work.

The scientific article structure is as follows: The Introduction sets the stage by addressing the significance of machine learning or deep learning-based anomaly detection in IoT, summarizing existing methods, and outlining research goals. Section 2 analyzes prior research, reviewing existing datasets created using approaches we described in Section 1. Section 3 describes the dataset creation methodology, data collection processes, and data processing. Section 4 discusses the overview of the dataset. Section 5 discusses the contributions of this study and proposes directions for future research, and Section 6 summarizes the key findings of the study.

## 2. Related Works on IoT Datasets

Numerous IoT security dataset contributions have been published in recent years. In this section, we present the characteristics of the existing datasets found in the literature to gain a better understanding of them.

### 2.1. Bot-IoT

This is a labeled dataset generated using the testbed environment at the Research Cyber Range lab of UNSW Canberra. For the purpose of simulating the network behavior of IoT devices, the Node-RED tool was utilized, and JavaScript code was built to replicate the behavior of IoT sensors such as pressure, temperature, and humidity. The ostinato tool was utilized to generate a massive amount of normal traffic. This dataset includes DDoS, DoS, OS and service scan, keylogging, and data exfiltration attacks scheduled to run at different times, with normal background traffic being constantly generated and collected in the same file. The dataset has more than 72 million records, including only 9543 instances of benign traffic. Also, the distribution of attack records is not uniform, with the information theft attacks having the least number of records. The Bot-IoT dataset has 43 network flow features consisting of 29 original features and 14 computed features. The dataset is publicly available in different formats, such as packet capture (PCAP) files, Argus files, and comma-separated values (CSV) files [24].

### 2.2. N-BaIoT

The Network-based Detection of IoT (N-BaIoT) focuses on botnet attack detection for IoT devices created at Ben-Gurion University in Israel from nine commercial IoT devices, such as cameras, doorbells, and thermostats. The devices were infected with two botnet malware: Mirai and Bashlite. The normal network traffic was gathered right after the device was installed. The raw network traffic was saved in PCAP format by using port mirroring on the switch. The dataset has 7,062,606 records, each containing 115 features describing various types of statistical properties of network traffic; however, precise counts for the proportion of malicious versus benign traffic are not provided. The data set is publicly available in CSV format [25,26].

### 2.3. Aposemat IoT-23

This is a labeled dataset created using three real IoT devices, consisting of 20 captures of malware and 3 captures of benign IoT device traffic at the Stratosphere Laboratory. The malicious scenarios include traffic from Mirai, Torii, Hajime, Hide and Seek, and others. The dataset contains more than 760 million packets and 325 million labeled flows, out of which 30.9 million flows are labeled as benign. The distribution of malicious labels is highly imbalanced; for instance, the label PartofAHorizontalPortScan alone accounts for approximately 213.9 million flows. In contrast, several labels, such as C&C-Mirai and C&C-File download, have only 2 and 53 flows, respectively. The dataset is available in two formats: a full compressed file of the entire dataset (consisting of the original PCAP and conn.log.labeled files) and a small-sized compressed file with only the net flows of each scenario (conn.log file) [27].

### 2.4. MedBIoT

This is a labeled behavioral IoT dataset that includes normal and malicious network traffic from actually deployed malware such as Mirai, BASHLITE, and Torii. The data is collected from 80 virtual IoT devices and 3 real devices. The real devices used in this dataset are the Sonoff Tasmota smart switch, TPLink smart switch, and TPLink light bulb, and the virtual devices include the lock, switch, fan, and light. The virtualization of IoT devices was implemented using Docker containers deployed using a Raspberry Pi to emulate the behavior of an IoT device. An automated execution approach was utilized for the simulation of benign behavior. A total amount of 17,845,567 network packets was captured, in which around 30% of this traffic was deemed and labeled as malicious, while 70% corresponds to legitimate network traffic. A total of 100 network traffic statistical features are calculated from the PCAP files captured, including features such as packet count, mean, and variance. The dataset is provided in raw PCAP files in two formats: Bulk: PCAP files are provided for each data source type (i.e., legitimate, Mirai, BASHLITE, and Torii), and fine-grained: PCAP files are provided for each data source, botnet phase, and device type [28,29].

### 2.5. CICIoT23

This dataset is created using 105 real IoT devices in an IoT lab at the Canadian Institute for Cybersecurity, using smart home devices such as cameras, sensors, and microcontrollers. It includes both benign and malicious network traffic consisting of 33 attacks classified into seven categories, such as DDoS, DoS, web-based, recon, spoofing, brute force, and Mirai. These attacks were carried out on the vulnerable IoT devices using other compromised IoT devices. The benign data traffic was captured in the absence of any attack scenario. The dataset consists of 46,686,579 flow records, each with 47 features, of which only 2.4% represent benign data. Also, the distribution of attack records in the dataset is imbalanced, with DDoS attack data having the largest number of records, whereas brute force attack records are the least represented. The CICIoT2023 dataset is available in two different file formats: PCAP and CSV [30].

### 2.6. Gotham Dataset 2025

This dataset is generated utilizing the Gotham testbed. The testbed includes 78 emulated IoT devices operating on various protocols, including CoAP, MQTT, and RTSP, configured as a Docker container or virtual machine integrated into a realistic network topology constructed in the GNS3 emulator. This dataset contains a variety of attack types, such as DoS, network scanning, Telnet brute force, CoAP amplification, and various stages of command and control communication. The dataset was collected in a distributed manner, capturing network traffic separately for each IoT device at the interface between the IoT gateway and the device. The dataset contains approximately 2.6 times more malicious traffic than benign. In total, the dataset contains 3.13 million packets of network traffic. However, the paper does not provide precise counts for the proportion of malicious versus benign traffic. The dataset has 23 features, including a label, and is publicly available in PCAP and CSV formats [31].

Despite the growing number of publicly available IoT intrusion detection datasets, a significant gap remains in the methods being used for network data generation. Many widely used datasets, such as Bot-IoT, rely heavily on scripted scenarios and synthetic network tools to simulate IoT behavior. Although the simulation-based approaches offer scalability, they often fail to capture the complexity and unpredictability of real-world IoT environments. In contrast, datasets like Aposemat-IoT23, CICIoT-23, and N-BaIoT used real IoT devices to gather network traffic, enhancing authenticity, but are costly, resource- and time-consuming, and difficult to scale. Recent datasets, such as Gothem 2025, have explored container-based emulation, yet they still depend on external network emulators, sacrificing the authenticity of the network traffic. In addition to this, existing IDS datasets contain an uneven distribution of network traffic across devices and a class data imbalance problem.

To address this, we introduce a novel method that uses an emulation-based approach to create virtual replicas of actual IoT devices. Our method differs from existing datasets in that it generates realistic, device-specific network traffic natively without relying on any external network emulators or traffic replay tools. Furthermore, we address the often-overlooked issue of class data imbalance by implementing the prefixing of the number of records to be extracted from each device included in the experiment. This ensures that the resulting dataset supports more accurate and unbiased training for anomaly-based NIDS, particularly those leveraging machine learning or deep learning techniques.

In Table 1, we present a brief description of the existing IoT IDS datasets, providing insights into the approach used and the number of devices, packet capture, and feature extraction tools.

## 3. Methodology

In this section a detailed explanation of the methodology utilized for implementing the proposed method for creation, data collection, and processing is presented. First, the experimental testbed is described in detail, followed by an explanation of the network traffic capture. Lastly, the procedures used for extracting flow-based features from the captured data and labeling the dataset are presented.

### 3.1. Physical Setup

**Figure 1:**
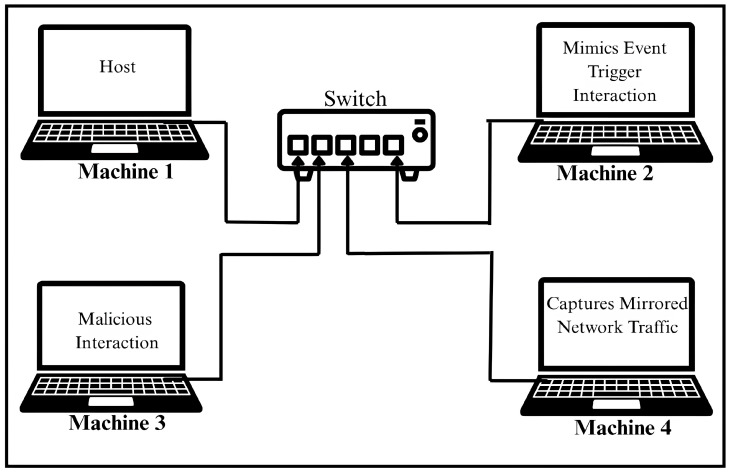
Architecture of experiment setup used for data collection.

The testbed used for implementation of the proposed method and capturing the dataset consisted of four physical machines running the Ubuntu OS, interconnected via Ethernet through a managed network switch. The switch was configured to enable port replication to facilitate passive traffic capture. In order to preserve a controlled and noise-free environment, the testbed was operated within an isolated local network environment, completely disconnected from the internet. All four machines were connected using a static topology that remained unchanged throughout data collection. No devices were added, reconfigured, or removed during data collection. The subsequent subsection details the roles of each machine deployed in the testbed. The visual representation of the testbed is illustrated in Figure 1.

Each of the four machines used in the testbed was assigned a specific role to replicate specific interaction patterns relevant to the realistic network traffic dataset generation. The functional role of each machine is described in detail below.

- Machine 1: Served as a host, running a virtual emulated IoT device within a QEMU-based environment.
- Machine 2: Served as an event-driven component to interact with the emulated virtual IoT device.
- Machine 3: Served as a malicious actor, performing unauthorized interactions.
- Machine 4: Served as a traffic-capturing tool.

### 3.2. Firmware Acquisition and Preparation of Virtual Environment

**Figure 2:**
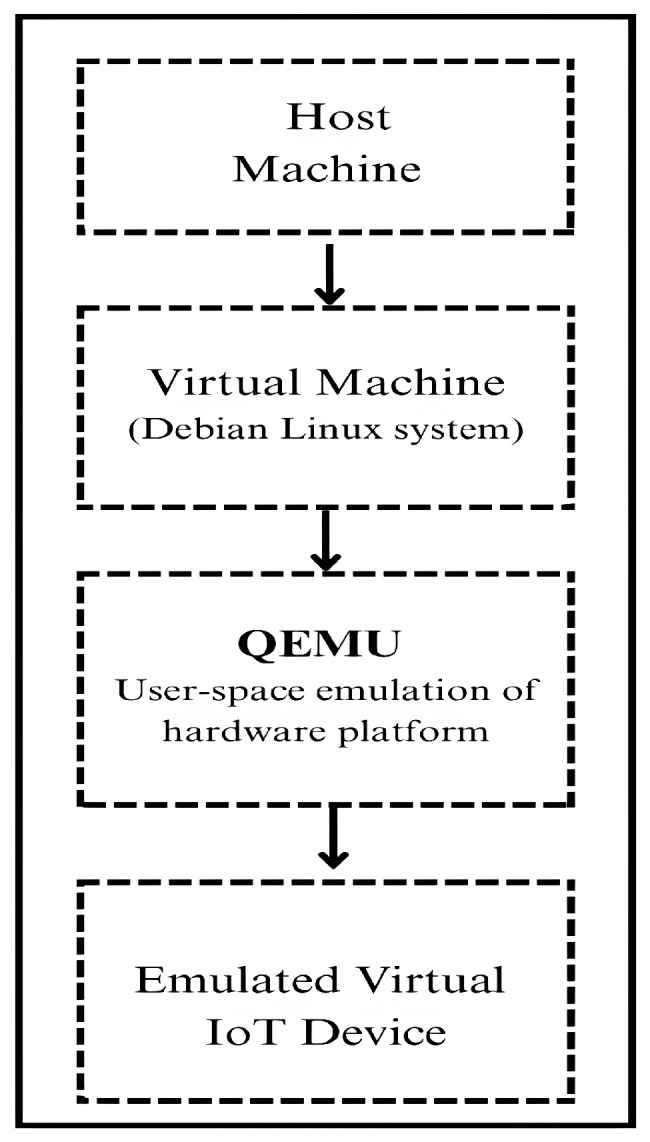
Layered architecture of QEMU-based IoT device emulation on host machine.

To create a virtual replica of an IoT device, official firmware images of IoT devices are collected from vendor websites. We then performed analysis of the collected file using the binwalk [32] tool to extract the file system, which helped in identifying the architecture (e.g., ARM, MIPS) and key binaries and services responsible for network communication. Based on these insights obtained, we initialized a corresponding minimal Debian Linux system using QEMU’s [33] user-mode emulation mode, which facilitates the execution of foreign-architecture binaries on the host machine. The obtained extracted firmware file is then integrated into this Debian environment and assigned a static IP address, effectively transforming the virtual emulated IoT device into a close replica of the original embedded device. The virtualization setup used for IoT device emulation is depicted in Figure 2.

For each firmware of the IoT device, we created a separate QEMU virtual machine on the same host machine, each running with its own architecture-specific minimal Debian-based Linux system. This setup allowed independent emulation of multiple devices, ensuring isolated traffic generation per device. Table 2 presents the list of emulated virtual IoT devices with details such as vendor name and device version.

### 3.3. Network Configuration

To enable realistic network traffic, a bridged network using a host-configured network bridge in combination with a tap (Terminal Access Point) interface was established. Specifically, we assigned an IP address to the virtual bridge, and then we allocated a tap device, each with its own IP address, for every emulated virtual IoT device. The tap interface acts as a virtual Ethernet port, allowing QEMU or similar emulators to send and receive packets as if it were a physical machine on the same local network (LAN). This setup enables the emulated virtual IoT device to send and receive realistic network traffic just like a real IoT device on a physical network.

**Figure 3:**
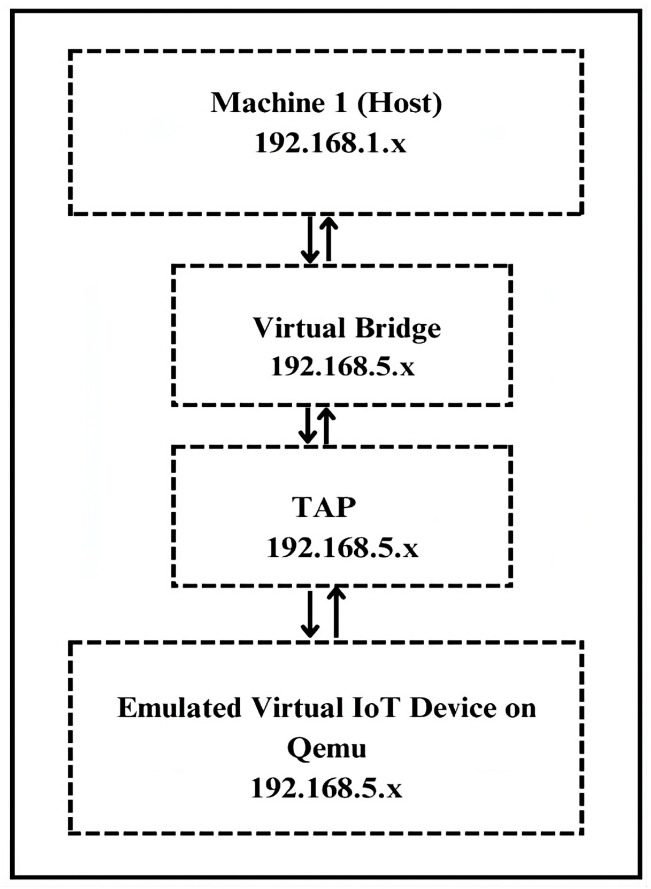
Network configuration with subnet isolation of host machine and emulated virtual IoT device.

Each physical and virtual machine was assigned a static IP address, as it helped in segregating traffic from the host systems and the virtual machines. The host machines operated within the 192.168.1.x subnet, while the virtual machine operated on the 192.168.5.x subnet. The virtual machine is enabled to communicate with other physical machines that share the same subnet as the host by employing static routing using the IP route utility with IP forwarding on the host machine. This configuration lets virtual machine packets traverse the host’s network stack, enabling it to communicate with other physical machines in the testbed. This arrangement bridges the isolated virtual subnet with the host’s physical network; the setup facilitates transparent, bidirectional communication between the virtualized environment and external physical nodes. The visual illustration of network architecture connecting the host machine (Machine 1) and the virtual IoT device with their respective subnets is presented in Figure 3.

### 3.4. Network Traffic Capture

The emulated virtual IoT devices replicate the network communication logic of real IoT devices without reproducing their functionality or sensor-driven operations. Therefore, to facilitate interaction between emulated virtual devices and physical machines deployed within the testbed, a script-driven approach was employed to generate both benign and attack traffic in a controlled manner. Both benign and attack sessions were executed sequentially and independently, with only one virtual IoT device active at a time. The benign traffic was captured first, followed by malicious data collection under similar network conditions.

The network traffic was captured in packets using tcpdump, running on Machine 4. To capture the traffic passively without affecting the device’s communication, network traffic was collected using port mirroring at the switch level for both benign and attack traffic. This ensured clean, unambiguous packet traces free from cross-device interference. The captured network traffic from all devices was collected in separate PCAP files for further processing, such as feature extraction, data cleaning, and labeling. The PCAPs were stored on Machine 4 device-wise, with benign traffic and attack traffic stored separately. Furthermore, attack traffic was categorized and stored according to its type and subtype. The network traffic was collected in two distinct stages, as explained in subsequent sections.

#### 3.4.1. Benign Traffic Generation

**Figure 4:**
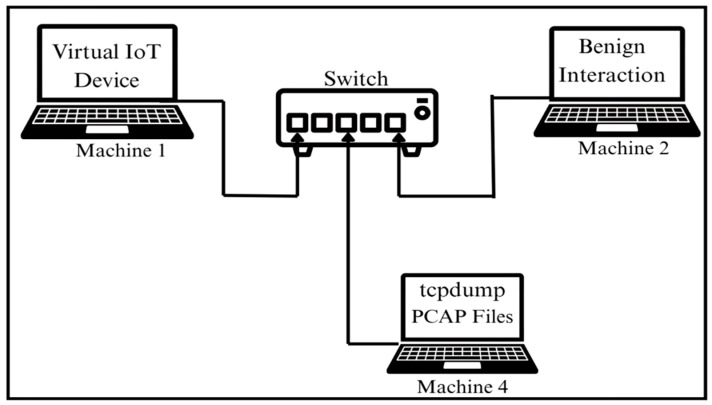
Workflow for benign interaction and traffic collection.

We designed a custom bash script for benign traffic designed to replicate event-driven interactions with emulated virtual IoT devices. Additionally, to eliminate fixed execution patterns, we intentionally designed the scripts with randomized execution of operations, timing, order, and payload structures. This randomness aimed to better reflect the unpredictable nature of real-world IoT behavior, where device communications are often event-driven. Moreover, in order to ensure consistency and reduce operational complexity, we only included those events in the script that were common across all devices, such as ping requests, periodic keep-alive signals, short bursts, and miscellaneous background communication. The script ran continuously on Machine 2 to interact with one active virtual IoT device at a time for over a period of 24 h to generate benign network data. The process of collecting benign data is visually illustrated in Figure 4.

#### 3.4.2. Malicious Traffic Generation

Malicious traffic was generated using a combination of various tools and custom bash scripts to simulate various cyberattacks. Similar to the benign network traffic, to avoid any fixed pattern in malicious network traffic, the attack scripts were designed with randomized patterns and timings. The execution time of attack scripts varied depending on the nature of each attack category. In Table 3, the attack types with the respective tools used to generate malicious network data are presented.

**Figure 5:**
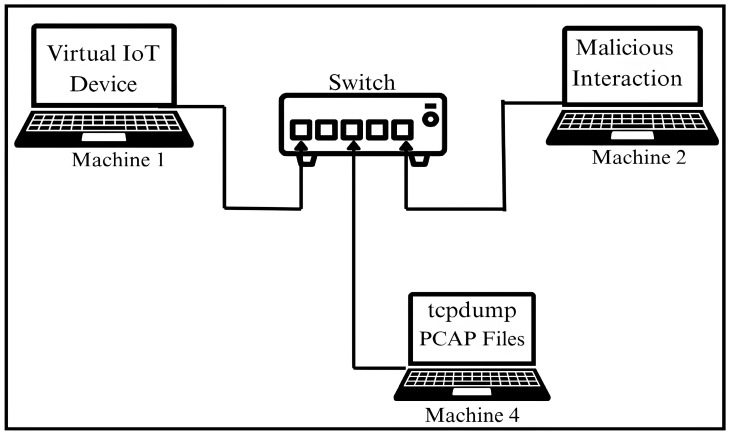
Workflow for malicious interaction and traffic collection.

Most attacks were launched from Machine 3, targeting one emulated virtual IoT device at a time. However, in the case of the exploitation attack, the connection was initiated from the emulated IoT device to Machine 3 to mimic a real-world scenario. The process of collecting malicious data is visually illustrated in Figure 5.

### 3.5. Feature Extraction, Data Cleaning, and Labeling

To automate the transformation of raw PCAPs into a clean, balanced, and labeled dataset, we developed separate Python scripts (version 3.9.22), each responsible for handling a distinct task. The script transformed one PCAP file at a time, starting from benign and then followed by malicious PCAP files.

#### 3.5.1. Script 1: Feature Extraction

The first step began by loading a PCAP file into the nfstreamer library. The nfstream is a Python framework designed for network traffic analysis that provides flow-based feature extraction from PCAP files. The nfstreamer was configured to perform statistical analysis on the PCAP file. The extracted features were structured into a Pandas DataFrame, where each row represented a unique network flow and each column captured a specific feature. The output of this stage was exported in a CSV file containing flow-level statistical data.

#### 3.5.2. Script 2: Elimination of Duplicate Rows

In the second step, the CSV file generated in the previous step was processed to identify and remove duplicate rows. This step ensured that only unique records are present in the dataset, helping to prevent bias in training machine and deep learning techniques. The resulting intermediate CSV containing only distinct network flows is then exported for further processing.

#### 3.5.3. Script 3: Data Cleaning and Labeling

In the third step, data cleaning and labeling were performed on the intermediate CSV file. The CSV was scanned to detect and remove any rows or columns with missing, null, or incomplete values. Once the data cleaning was completed and an intermediate CSV file with reliable features was exported, the script then assigned a label to each record according to its traffic type. In particular, benign records were labeled as benign, whereas malicious records were labeled using a structured format that combined the attack category and subtype. The labeled records were initially stored as CSV files device-wise and later merged into two consolidated CSV files—one for benign and one for malicious traffic.

## 4. EmuIoT-VT Dataset Overview

This section provides an overview of the dataset along with the data balancing approach adopted for its construction. This section also provides the details about the organization, data format, and features present in the dataset. The dataset includes several cybersecurity attacks such as DoS, brute force, exploitation, and reconnaissance. The label field can take nine different values, including benign, mal_dos_synflood, mal_dos_icmpflood, mal_dos_tcpflood, mal_recon_osscan, mal_recon_portscan, mal_recon_versionscan, mal_Reverse_Shell, and mal_BruteForce. To eliminate the noise, unintended traffic, or any external interference, all network traffic was captured in an isolated local environment completely disconnected from the internet.

### 4.1. Dataset Balancing

To retain all network traffic data while ensuring that the final processed dataset used for training and evaluation maintained an equal distribution of benign and malicious instances, the dataset was balanced during the feature extraction phase. In order to maintain uniform representation of network data across all devices, classes, and subclasses, and to avoid bias in the resulting dataset, we decided to prefix the number of network records to be extracted from each captured file.

**Figure 6:**
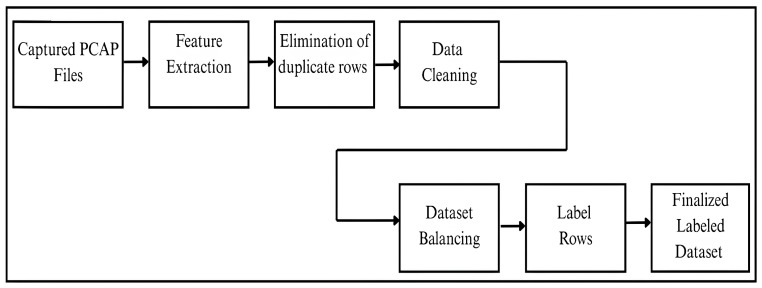
Pipeline for transforming raw PCAP into a balanced, labeled dataset.

The steps to transform raw PCAP files into CSV files are detailed in Section 3.5. Once the cleaned CSV file was obtained, the same script counted the number of rows in the intermediate file. If the row count exceeded the predefined threshold, the excess rows were excluded. Following this, a label corresponding to the traffic type was assigned to each row. In particular, a target of 2000 records was set for both benign and malicious traffic, respectively, from each device. Malicious records were proportionally allocated across four primary attack categories—DoS, reconnaissance, brute force, and reverse shell—with 500 records assigned to each category. Each category was further divided into three subcategories (e.g., mal_dos_synflood, mal_dos_icmpfood, and mal_recon_portscanning), yielding approximately 167 records per subcategory. As a result, the dataset supports both binary (benign vs. malicious) and multi-class (attack-type-specific) classification tasks with strong consistency across devices and inter-classes. The steps of converting a PCAP file into a balanced CSV-formatted dataset are depicted in Figure 6.

### 4.2. File Format and Organization

The finalized dataset was delivered in a structured format consisting of two CSV files: one for benign traffic and one for malicious traffic. The dataset contains a total of 82 features, including source and destination identifiers, protocol information, flow timing, traffic volume, statistical patterns, and TCP flag behavior. It also incorporates application-level metadata. Each record is labeled as benign or malicious. The CSV file contains a total of 28,000 records, comprising 14,000 instances of benign flows and 14,000 instances of malicious records.

## 5. Discussion

This work makes two major contributions: the first is the introduction of a new emulation-based method for realistic IoT device network traffic generation, and the second is the construction of a realistic, labeled dataset—EmuIoT-VT generated through implementing the proposed method. The objective was to develop an approach that is reproducible, scalable, and generates realistic network data without relying on physical IoT hardware, containerized abstractions, traffic replay tools, or network simulators. The proposed method creates virtual replicas of IoT devices that generate network traffic through their network stack that closely resembles that generated by the actual device under real-world network conditions.

Unlike several existing works that have used an emulation-based approach to create virtual IoT devices using high-level scripting or containers, our framework does not emulate device behavior externally. Instead, each emulated device operates using TAP and bridged networking, where all traffic originates from the internal execution of its firmware. No external traffic generators or network simulators were used.

The dataset was generated in a fully offline, isolated environment to capture raw, uncontaminated network traffic generated solely by the emulated IoT devices. This isolated environment helped in the accurate labeling of benign and malicious flows.

A defining feature of this work is the use of the balance by design strategy, as most publicly available IoT datasets suffer from a severe class imbalance problem. EmuIoT-VT avoids the class data imbalance problem by balancing the dataset during the feature extraction phase. This balance was achieved by prefixing the number of records to be kept, not by applying synthetic resampling techniques afterward.

While EmuIoT-VT offers several advantages, there are opportunities for future extension. First, to expand the dataset by incorporating a broader range of IoT devices from the same or different domains. Second, future versions may introduce internet exposure and multi-device interactions. Additionally, future studies will validate the EmuIoT-VT dataset by training baseline machine learning and deep learning models.

## 6. Conclusions

During the literature review, it was observed that despite having many publicly available IoT IDS datasets, a significant gap remains in the methods being used for network data generation. Moreover, the existing datasets suffer from class imbalance problems in training data, which hinders the performance of anomaly-based NIDS that leverage machine learning or deep learning techniques. As a result, despite achieving high accuracy, they often struggle in detecting minority-class data.

To address the aforementioned issues, in this study, a new method was proposed leveraging an emulation-based approach for creating a virtual replica of an IoT device in a virtual environment capable of producing real-network traffic patterns. The proposed method bridges the gap between controlled experimentation and real-world IoT deployments.

Through the implementation of the proposed method, a new network dataset was constructed by collecting network traffic data in an isolated local network without any internet connectivity. This dataset was designed to address the limitations observed in existing datasets and contains uniform data across devices as well as across classes and subclasses, making it more suitable for anomaly-based NIDS using machine learning or deep learning techniques. Additionally, the proposed method and its implementation were designed to be compatible with those that utilize real devices, thereby offering a resource- and cost-efficient solution.

This work presented a novel methodology for generating realistic IoT network traffic using a firmware-level emulation approach. By executing actual IoT device firmware in QEMU, we created virtual replicas capable of producing authentic network traffic patterns. Unlike simulation-based or synthetic traffic generation methods, this approach offers higher fidelity by preserving the native protocol behavior of the device firmware.

Using this emulation setup, we constructed EmuIoT-VT, a new dataset comprising 28,000 labeled network flows, evenly split between benign and malicious traffic. Attacks include denial-of-service, brute force, reconnaissance, and exploitation, while benign traffic was generated through scripted interactions with emulated devices. The dataset was captured in a fully isolated network environment, ensuring clean, noise-free traffic that supports precise labeling and reproducibility.

Additionally, EmuIoT-VT was designed with balanced class distributions across traffic types and attack subcategories to mitigate the effects of class imbalance commonly found in existing datasets. The dataset contains 82 features extracted from raw PCAP files, making it suitable for training and evaluating both traditional and machine- or deep learning-based intrusion detection models.

The proposed method and dataset offer a scalable and cost-effective alternative to real-device testbeds, enabling researchers to generate clean and interpretable IoT traffic without physical hardware dependencies.

## Figures and Tables

**Table 1:** Summary of Existing IoT Datasets.

Dataset	Year	Approach Used	Number of Devices Used	Packet Capture and Feature Extraction Tool	Number of Features	Data Imbalance
Bot-IoT	2018	Simulation	5	Packet capture: tshark Feature extraction: argus	43	Yes
N-BaIoT	2018	Real Device	9	Packet capture: wireshark Feature extraction: wireshark	115	Precise count not provided
Aposemat IoT-23	2020	Real Device	3	Packet capture: zeek Feature extraction: zeek	-	Yes
MedBIoT	2020	Mixed	3 real and 80 virtual devices	Packet capture: tcpdump Feature extraction: splunk	100	Yes
CICIoT23	2023	Real Devices	105	Packet capture: wireshark, tcpdump Feature extraction: dpkt package	47	Yes
Gotham2025	2025	Emulation	78	Packet capture: tcpdump Feature extraction: tshark	23	Yes
Proposed EmuIoT-VT	2025	Emulation	7	Packet capture: tcpdump Feature extraction: nfstream	82	No

**Table 2:** List of virtual IoT devices with their details used to produce the dataset.

Number	Virtual IoT Device with Details
1	D-Link DCH-S160 wi-fi water sensor
2	D-Link home siren DCH-S220
3	ToToLink T6 AC1200 dual band smart home wi-fi router
4	ToToLink EX200 wireless N300 range extender
5	D-Link wi-fi smart plug DSP-W110
6	D-Link Dcs-932l day/night cloud camera
7	Lametric smart clock

**Table 3:** Attack types and tools used to generate malicious interaction and network traffic data.

Name	Attack Type	Tools
DoS	tcp flood	hping3 [34]
	syn flood	
	icmp ping flood	
Reconnaissance	port scan	nmap [35]
	OS scan	
	version scan	
Brute Force	dictionary brute force	hydra [36]
Exploitation	reverse shell	custom bash script

## Data Availability

All data relevant to the research are provided at https://zenodo.org/records/16727174?token=eyJhbGciOiJIUzUxMiJ9.eyJpZCI6IjAzOThlOGQ2LTA1MzUtNDJlOC1hMDQ1LWRlMGQ0ZDg2NDdiNyIsImRhdGEiOnt9LCJyYW5kb20iOiI0NDA4OWU1OWNhN2Y2MDZkNjliNDNkMTIwNWNkMGIyOCJ9.J2dxr8x33COoMbPx6B2SWRBscTkFCpaU4W6N6ZnbR4Sk93xxsJdLg4EQAeWDNTbkTn3veUWmXfQPNuqDkI8ZUw (accessed on 3 August 2025).

## Abbreviations

The following abbreviations are used in this manuscript:

OS	Operating System
DDoS	Distributed Denial-of-Service
DoS	Denial-of-Service
NIDS	Network Intrusion Detection System
